# Therapy-based strategies to support tummy time in infants post-hospital discharge: A scoping review protocol

**DOI:** 10.1371/journal.pone.0324435

**Published:** 2025-05-28

**Authors:** Ketaki Inamdar, Sonia Khurana

**Affiliations:** 1 Department of Human Performance, Physical Therapy, West Virginia University, Morgantown, West Virginia, United States of America; 2 Ellmer College of Health Sciences, Macon & Joan Brock Virginia Health Sciences at Old Dominion University, Norfolk, Virginia, United States of America; Sri Ramachandra Institute of Higher Education and Research (Deemed to be University), INDIA

## Abstract

Tummy time is essential for infant development, yet many caregivers face significant challenges with adherence due to behavioral and contextual barriers. While numerous tummy time interventions exist, a limited understanding of their behavioral components hinders effective replication and implementation. This scoping review aims to identify multidisciplinary interventions used to promote tummy time in infants aged 0–12 months, evaluate their impact on adherence, developmental and health outcomes, and examine the behavior change techniques employed using the Theoretical Domains Framework (TDF). Following the Joanna Briggs Institute (JBI) methodology, a comprehensive search will be conducted in MEDLINE (PubMed), Web of Science Core Collection, CINAHL (EBSCOhost), ClinicalTrials.gov, and gray literature sources for relevant studies published in English between January 1994 and January 2025. Eligible studies will include experimental research involving infants aged 0–12 months who received targeted tummy time interventions following hospital discharge across various early intervention settings. Data extraction will be performed by two independent reviewers using a customized tool, with results presented as a narrative summary, tabular form, or diagrams, as appropriate. Findings from this review will inform the development of behaviorally grounded, clinically feasible tummy time strategies that are better aligned with caregiver needs.

## Introduction

Tummy time, also known as prone play or prone positioning, refers to the practice of placing infants on their stomach while awake and supervised [1]. Tummy time is widely recognized for its role in supporting early motor development in infants by promoting head control, accelerating motor milestone achievement, preventing positional plagiocephaly, and reducing body mass index [2]. Additionally, tummy time affords opportunities for environmental exploration and social interaction, which may foster cognitive and social development [3,4]. Given these benefits, organizations such as the World Health Organization (WHO) and the American Academy of Pediatrics (AAP) strongly advocate for incorporating tummy time into an infant’s daily routine, beginning at birth with 3–5 minutes, and progressively increasing to 60 minutes per day by three months of age [1,5].

Despite clear guidelines and well-documented benefits, many caregivers struggle to implement tummy time consistently. Common barriers include low parental self-efficacy, competing caregiving responsibilities, infant discomfort and distress, and a lack of positive caregiving experience during tummy time [6]. These challenges underscore the complexity of tummy time implementation; it requires active caregiver engagement, is influenced by multiple external factors (e.g., family routines, adult assistance), and depends on the infant’s response, which can vary widely. It demands ongoing effort, reinforcement, and adaptability from the caregivers [7]. Given these difficulties, promoting tummy time adherence requires more than just providing educational resources — it necessitates targeted behavior change strategies. Although numerous interventions have been developed to support tummy time, [8–10], there is a limited understanding of the behavioral mechanisms that drive their success. Existing research primarily focuses on the association of tummy time with developmental outcomes [2–4] and implementation barriers [6,11,12] but has not systematically examined how existing tummy time interventions address caregiver challenges or promote adherence.

**Fig 1:**
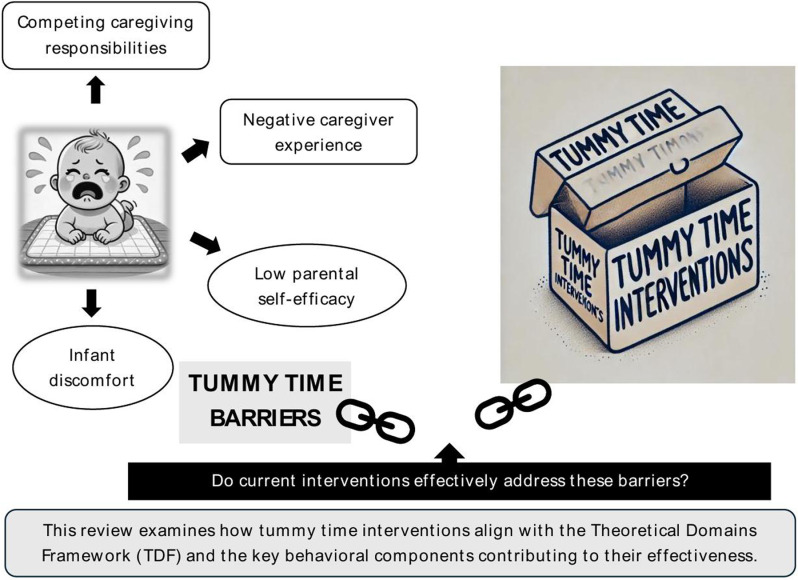
Conceptual overview illustrating this review’s aim to highlight the gap between reported barriers to tummy time and existing intervention strategies.

The Theoretical Domains Framework (TDF), provide a structured approach to understanding behavior change by synthesizing multiple theories into key domains, such as knowledge, beliefs about capabilities, social influences, and environmental context etc. [13] These domains are said to influence therapeutic decisions and have been widely applied in pediatric therapeutic interventions to improve therapist- and caregiver-led practices. For example, the TDF has informed strategies for implementing parent-delivered interventions for infants at risk of cerebral palsy [14], identified factors influencing therapeutic goal-setting in children with acquired brain injury [15], and retrospectively analyzed the theoretical underpinnings of existing safe sleep interventions for infants [16]. Applying the TDF to tummy time interventions can provide critical insights into the behavioral mechanisms that drive caregiver engagement and intervention effectiveness. To address this gap, this scoping review will identify existing tummy time interventions and retrospectively code them using the TDF to determine the key behavioral components that influence intervention success (Fig 1). By synthesizing behavior change techniques used in tummy time interventions, this review aims to establish a clearer understanding of “what works,” “how it works,” and how specific behavioral strategies impact caregiver adherence and infant health outcomes.

A preliminary search of JBI Evidence Synthesis, the Cochrane Database of Systematic Reviews, PubMed, PROSPERO, and Epistemonikos was conducted in March 2024, identifying two systematic reviews on tummy time [2,12]. One review was published in 2017 [12]; and examined demographic, environmental, and behavioral factors influencing tummy time. The other review was published in 2020 [2], and assessed associations between tummy time and health outcomes using observational and intervention studies. However, both reviews focused exclusively on healthy infants and did not analyze the behavioral strategies embedded within tummy time interventions. In contrast, this scoping review will provide novel insights by evaluating the design and implementation of tummy time interventions, identifying key ingredients for success, and including both healthy infants and those with medical conditions such as prematurity or genetic syndromes.

### Review question(s)

1. What types of multidisciplinary interventions currently exist to promote tummy time in infants aged 0–12 months?
2. What is the impact of these interventions on tummy time adherence, developmental domains, and health outcomes?
3. How do these interventions align with the Theoretical Domains Framework (TDF), and what key behavioral components contribute to their effectiveness?

### Inclusion criteria

#### Participants:

This review will consider studies that include infants aged 0–12 months. Eligible populations will include typically developing full-term infants, infants born preterm (<37 weeks of gestation), and infants with diagnosed medical or genetic conditions such as Down syndrome, cerebral palsy, perinatal stroke, or congenital heart disease. Studies with longitudinal data collection (e.g., follow-ups at 3, 9, 12, 24, or 36 months) will be eligible only if the tummy time intervention was implemented within the first year of life. For studies with broader age ranges, inclusion will require that at least 90% of participants are aged 0–12 months, unless the study includes sub-group analyses for infants within this age range. In such cases, the study will be considered if the subgroup of infants aged 0–12 months is clearly identifiable and reported in sufficient detail.

#### Concept:

This review will consider all studies that implement a focused tummy time intervention provided during the first year of life after hospital discharge. Tummy time interventions are operationally defined as “structured programs, activities, or strategies designed to promote or facilitate prone positioning (tummy time) in infants while awake and supervised. These interventions may involve caregiver education, environmental modifications, behavioral strategies, positioning aids, or guided play aimed at increasing tummy time duration, improving infant tolerance, and/ or supporting developmental outcomes”. Studies focusing on prone positioning during sleep or within the Neonatal Intensive Care Unit (NICU) will be excluded, as prone positioning in the NICU serves a different clinical purpose. NICU-based prone positioning is primarily used to improve physiological stability in at-risk infants and requires specialized training that cannot be replicated by untrained clinicians or caregivers [17,18]. Additionally, tummy time is frequently included as a component of broader early intervention [19] or physical activity promotion programs [20]. Such studies will only be included if they provide detailed descriptions of tummy time-specific guidelines or interventions. Multicomponent studies without clear tummy time guidelines will be excluded, as it would be difficult to isolate the effects of tummy time from other components and systematically map intervention elements to the TDF. Tummy time interventions may be provided by any member of the pediatric therapeutic team, including nurses, physical therapists, occupational therapists, special education therapists, pediatric rehabilitation researchers, or caregivers.

Outcome measures for this review will include tummy time adherence (e.g., duration, tolerance), developmental measures of gross motor skills (e.g., head control, milestone attainment, locomotion), fine motor skills (e.g., object exploration, upper extremity muscle activation), cognitive abilities (e.g., problem-solving behaviors, executive functioning), and social and language development (e.g., parent-child interaction). Additional outcome measures will assess broader health indicators, including obesity and fitness (e.g., adiposity, body mass index, ponderal index, bone health), psychosocial factors (e.g., resilience, self-efficacy, mental health), and specific conditions such as plagiocephaly and torticollis. These outcomes may be assessed using standardized developmental assessments such as the Bayley Scales of Infant Development, Alberta Infant Motor Scale, or Test of Infant Motor Performance; questionnaires such as tummy time recall, milestone surveys, or physical activity questionnaires; qualitative tools including behavioral coding or clinical observations; medical reports such as lab tests for body composition and bone health or brain scans; and objective methods including wearable sensors, surface electromyography, motion capture, or electroencephalography. Studies will be excluded if they focus solely on physiological parameters such as heart rate and oxygenation or if they examine only caregiver outcomes (e.g., caregiver self-efficacy) without measuring infant-related outcomes. All outcomes can be reported at any time between birth and 60 months of life.

#### Context:

The context for this review encompasses all settings outside the NICU, such as center-based programs, hospital or rehabilitation centers, outpatient clinics, daycare or preschool programs, or home-based settings. These settings may be located in any geographic region.

#### Types of sources:

This scoping review will include all experimental study designs, such as randomized controlled trials, non-randomized controlled trials, quasi-experimental studies, pre-post studies, case-control studies, case reports, and case series. Our preliminary search indicated no existing systematic reviews specific to this topic; however, related systematic reviews will be reviewed to identify relevant primary studies. Gray literature, including theses, dissertations, and peer-reviewed conference papers will also be considered. In cases where the same data is reported in multiple publications, the primary article will be used. However, if different data subsets (e.g., distinct infant populations or separate outcome timelines) are published separately, both articles will be included with a note on data overlap. Qualitative studies, narrative reviews, non-peer-reviewed conference publications, and conference abstracts without accompanying full papers will be excluded.

## Methods

The proposed scoping review will be conducted in accordance with the JBI methodology for scoping reviews [21] and, reported in line with the Preferred Reporting Items for Systematic Reviews and Meta-Analyses extension for Scoping Reviews (PRISMA-ScR) [22]. This protocol has been registered in Open Science Framework (https://osf.io/bfeh4).

### Search strategy

The search strategy will aim to identify both published and unpublished papers, including theses and dissertations. An initial limited search of MEDLINE (via PubMed) and Google Scholar was undertaken to identify relevant articles on the topic. The text words contained in the titles and abstracts of these articles, and the index terms used to describe the articles, were used to develop a comprehensive search strategy for MEDLINE via PubMed (S1 Table). The search strategy, including all identified keywords and index terms, will be adapted for each included database. Additionally, gray literature and the reference lists of all included studies will be screened for additional sources of evidence.

Articles published in or translated into English will be included, as English is the primary language of the research team. Studies published from January 1994 to January 2025 will be included, as 1994 marks the inception of the Back to Sleep campaign, which significantly influenced infant sleep positioning and motor development research, making earlier studies less relevant to current practices.

The databases to be searched include MEDLINE (PubMed), Web of Science Core Collection, CINAHL (EBSCOhost), and ClinicalTrials.gov. For gray literature, Google Scholar will be used to identify peer-reviewed journal articles that may not be indexed in traditional databases. Following the approach outlined by Haddaway et al., the first 200–300 search results will be screened for relevance [23]. Additionally, ProQuest Dissertations & Theses Global will be searched to identify relevant doctoral and master's theses, and the reference lists of all included studies will be screened for additional sources of evidence.

### Study/Source of evidence selection

Following the search, all identified records will be collated and uploaded into Rayyan (Rayyan Systems, Cambridge, MA, USA), an AI-assisted collaboration tool to conduct literature reviews, and duplicates will be removed. Titles and abstracts will then be screened by two independent reviewers for assessment against the inclusion criteria for the review. The full text of selected citations will then be reviewed in detail against the inclusion criteria by the same two reviewers. Reasons for excluding sources of evidence at the full-text stage will be recorded and reported in the scoping review. Any disagreements that arise between the reviewers at each stage of the selection process will be resolved through discussion or, if needed, with input from a subject expert. The results of the search will be reported in full in the final scoping review and presented in a PRISMA flow diagram [24]. No critical appraisal of the included sources of evidence is planned.

### Data extraction

Data will be extracted from papers included in the scoping review by two independent reviewers using a data extraction tool developed by the reviewers. To address the *first research question*, extracted data will include author(s), study location and setting, study design, sample size, participant demographics, medical diagnoses (if applicable), and intervention name. Intervention details will be further described using the Template for Intervention Description and Replication (TIDieR), which outlines key components such as intervention name, rationale, materials, delivery methods, setting, timing, tailoring, and modifications [25]. For the *second research question*, extracted data will include the outcome domain (e.g., gross motor skills), assessment time points, changes over time, and group differences. To address the *third research question*, included tummy time interventions will be mapped to the 14 domains of the Theoretical Domains Framework (TDF), including knowledge, skills, social/professional role and identity, beliefs about capabilities, beliefs about consequences, motivation and goals, memory, attention, and decision processes, environmental context and resources, social influences, emotion, reinforcement, intentions, goals, and behavioral regulation [13]. Codes will first be independently assigned by two researchers and then re-reviewed and discussed to confirm final domain allocation. A draft extraction tool is provided (see S2 Table). This tool will be modified and revised as necessary during the process of extracting data from each included paper. Modifications will be detailed in the full scoping review. Any disagreements that arise between the reviewers will be resolved through discussion or with a subject expert.

### Data analysis and presentation

The extracted data will be presented in tabular or diagrammatic format in a manner that aligns with the objectives of the review. Specifically, demographic trends will be illustrated using diagrams to enhance visualization. Intervention details, outcome measures, and TDF domain mapping will be organized in tabular format. To analyze intervention effectiveness, quantitative data will be summarized by reporting p-values, means, and medians for within- and between-group comparisons, along with effect sizes when available. A narrative summary will accompany the tabulated results, describing how the findings address the research questions. Additionally, the narrative will highlight the strengths and limitations of the interventions based on the extracted data and provide insights into potential areas for future research. The TDF will guide data analysis by evaluating how different interventions align with the psychological and contextual factors influencing behavior change. Study timeline: A) Data collection will be completed – April 2025, B) Analysis will begin May 2025 and C) Results are expected July 2025.

## Supporting information

S1 Table PubMed search strategy. (DOCX)

S2 Table Draft data extraction instrument. (DOCX)
