# Supplementary material for: Therapy-based strategies to support tummy time in infants post-hospital discharge: A scoping review protocol
Source: PLoS One. 2025 May 28;20(5):e0324435. doi: 10.1371/journal.pone.0324435 (PMC12118841; doi:10.1371/journal.pone.0324435)
Supplement: S1 Table — (DOCX) [file pone.0324435.s001.docx]

**Table S1. PubMed search strategy**

| **Search** | **Query** | **Records retrieved** |
| --- | --- | --- |
| #1 | ("infant"[MeSH Terms] OR "infant"[All Fields] OR "infants"[All Fields] OR "infant s"[All Fields]) AND 1994/01/01:2025/01/28[Date - Publication] | 922,404 |
| #2 | ("tummy time"[All Fields] OR "prone play"[All Fields] OR "prone position*"[All Fields]) AND 1994/01/01:2025/01/28[Date - Publication] | 10,175 |
| #3 | ("intervention*"[All Fields] OR "effect*"[All Fields] OR "efficacy*"[All Fields] OR "result*"[All Fields] OR "achieve*"[All Fields] OR "consequence*"[All Fields] OR "outcome*"[All Fields] OR "conclusion*"[All Fields]) AND 1994/01/01:2025/01/28[Date - Publication] | 18,477,791 |
| #3 | #1 AND #2 AND #3 | 1,031 |
| #4 | #3 AND english"[Language] | 956 |
| Limited to date of publication (1994/01/01:2025/01/28) and language (English) applied | |  |

*Last search conducted on January 28, 2025
