# Supplementary material for: Therapy-based strategies to support tummy time in infants post-hospital discharge: A scoping review protocol
Source: PLoS One. 2025 May 28;20(5):e0324435. doi: 10.1371/journal.pone.0324435 (PMC12118841; doi:10.1371/journal.pone.0324435)
Supplement: S2 Table — (DOCX) [file pone.0324435.s002.docx]

**Table S2. Draft data extraction instrument**

| **Information Type** | **Data Extracted** |
| --- | --- |
| *Study Description and Participant Characteristics*  Title  Citation  Journal  Study location  Study aim  Total sample size  Sample size per group  Population diagnosis  Population gestational age (if provided)  Population chronological age range or mean  Population adjusted age (if applicable)  Age at the start of intervention  Age at the end of intervention  *Template for Intervention Description and Replication (TIDieR) for both intervention and control groups (when applicable)*  Name  Why  What (Materials)  What (Procedures)  Who Provided  How  Where  When and How Much  Tailoring  Modifications  How Well (Planned Fidelity Assessment)  How Well (Actual Fidelity Assessment)  *Outcome Measures*  Name of outcome measure  Metric  Time point of outcome assessment  Baseline scores  Post-intervention scores  Change in scores over time (summary measures, p-value, and effect size when provided)  *Theoretical Domains Framework*  Knowledge  Skills  Social/professional role and identity  Beliefs about capabilities  Optimism  Beliefs about consequences  Reinforcement  Intentions  Goals  Memory, attention, and decision processes  Environmental context and resources  Social influences  Emotion  Behavioral regulation |  |
